# Case Report: Imaging immune checkpoint inhibitor-induced yin-yang effects in the brain

**DOI:** 10.3389/fimmu.2023.1199282

**Published:** 2023-06-02

**Authors:** K. F. Bol, E. Peeters, C. M. L. van Herpen, H. Westdorp, E. H. J. G. Aarntzen

**Affiliations:** ^1^ Medical Oncology, Radboudumc, Nijmegen, Netherlands; ^2^ Medical Imaging, Radboudumc, Nijmegen, Netherlands

**Keywords:** PET/CT imaging, immune checkpoint inhibitors, CD8+ T-cells, toxicity, melanoma

## Abstract

**Background:**

Treatment with immune checkpoint inhibitors (ICI) can induce durable responses in cancer patients, but it is commonly associated with serious immune-related side effects. Both effects are suggested to be mediated by CD8+ T-cell infiltration. Whole body CD8+ T-cell distribution can be visualized by PET imaging of a 89Zr-labeled anti-humanCD8a minibody, currently investigated in a phase 2b trial.

**Main body:**

An adult patient diagnosed with metastatic melanoma developed ICI-related hypophysitis after two courses of combined immunotherapy (ipilimumab (3 mg/kg) and nivolumab (1 mg/kg) at 3 weeks interval). On a [^89^Zr]Zr-crefmirlimab berdoxam PET/CT scan, made 8 days before clinical symptoms occurred, increased CD8+ T-cell infiltration in the pituitary gland was detected. Simultaneously, tracer uptake in a cerebral metastasis was increased, indicating ICI-induced tumor infiltration by CD8+ T-cells.

**Conclusions:**

The observations in this case report underscore the role of CD8+ T-cell in non-tumor tissues in ICI-related toxicity. In addition, it illustrates a potential role for molecular imaging by PET/CT for investigation and monitoring of ICI-induced effects.

## Highlights

Non-invasive CD8+ T-cell tracking in patients visualizes CD8+ T-cell infiltration in tumor, as well as in healthy organs. This novel technology is under investigation to assess its predictive potential, but may also allow early monitoring of immune checkpoint inhibitor-related toxicity.

## Introduction

Immune checkpoint inhibition has revolutionized the treatment of metastatic melanoma patients (1). Combined targeting cytotoxic T-lymphocyte antigen-4 (CTLA-4) and programmed death ligand-1 (PD-L1) maximizes cytotoxic (CD8+) T-cell activation, resulting in higher objective response rates and an improved median overall survival as compared to checkpoint inhibitor monotherapy (2). Increased T-cell activation is also associated with immune-related adverse events, and severe toxicities occur frequently under combination treatment (1, 3). Immune-related adverse events can be life-threatening and may warrant early and aggressive immunosuppressive treatment, but tools for early detection are lacking (4). In this report, we present a case with immune-related hypophysitis, which was detected with PET imaging of a ^89^Zr-labeled anti-hCD8α minibody, with the product name [^89^Zr]Zr-crefmirlimab berdoxam (5), to non-invasively track CD8+ T-cells, 8 days before clinical presentation.

## Case presentation

An adult male patient 73 years of age was diagnosed with metastatic melanoma localized in the brain, gallbladder, ampulla of Vater, and left maxillary sinus, as determined by contrast-enhanced CT scan of chest and abdomen, and dynamic contrast-enhanced MRI of the brain. The patient had one solitary brain metastasis of which the largest diameter was 32 mm. Lactate dehydrogenase was normal and ECOG performance score was 1. A *BRAF^V600E^* mutation was present but patient agreed to the preferred first-line of treatment with combined immunotherapy consisting of ipilimumab (3 mg/kg) and nivolumab (1 mg/kg) at 3 weeks interval. Patient consented to participate in study NCT05013099, a phase 2b study investigating PET/CT scans with [^89^Zr]Zr-crefmirlimab berdoxam, a radiolabeled anti-hCD8α minibody. PET/CT scans were made at baseline and after two courses of combination treatment, to evaluate treatment induced changes in CD8+ T-cell distribution *in vivo*. A week after the 3^rd^ course of immunotherapy patient presented with general malaise, weakness, headache, decreased appetite and feeling cold. Laboratory evaluation showed mild hyponatremia (sodium 127 mmol/l; reference, 135-145), an elevated C-reactive protein (53 mg/l; reference, <10), and decreased hormone levels of multiple endocrine axes (cortisol 0.13 µmol/l at 9:20 a.m.; reference, 0.19-0.55: adrenocorticotropic hormone 1.4 pmol/l; reference 1.6-13.9: TSH 0.26 mE/l; reference, 0.27-4.20: FT4 5.2 pmol/l; reference, 10.0-23.0: FSH 3.5 E/l; reference 1.5-12: LH 0.79 E/l; reference, 1.7-8.6: testosterone <0.42 nmol/l; reference, 10.5-37: prolactin 17 mE/l; reference, 86-320). Insulin-like growth factor, lactate dehydrogenase and potassium levels were normal. Physical examination revealed no abnormalities except for a decreased blood pressure of 124/65 mmHg (previous blood pressures 160-180/80 mmHg) with a pulse of 91/min. The clinical diagnoses of immune checkpoint inhibitor-related hypophysitis affecting multiple endocrine axes was made. Hormonal replacement therapy with hydrocortisone (20 mg/day) and levothyroxine (50 µg/day, later increased to 75 µg/day) was initiated after which the symptoms improved rapidly. The fourth course of combined immunotherapy was not given due to an adrenal crisis despite start of hormonal treatment. Patient was admitted with symptoms of headache and fever and treated with high dose hydrocortisone after which he recovered quickly. An intercurrent problem triggering the adrenal crisis was not diagnosed and the patient was discharged from the hospital after 4 days. Planned tumor evaluation 13 weeks after start of ipilimumab plus nivolumab showed stable disease per RECIST 1.1, including the cerebral metastasis, and no new lesions. Patient continued nivolumab monotherapy as planned.

**Figure 1:**
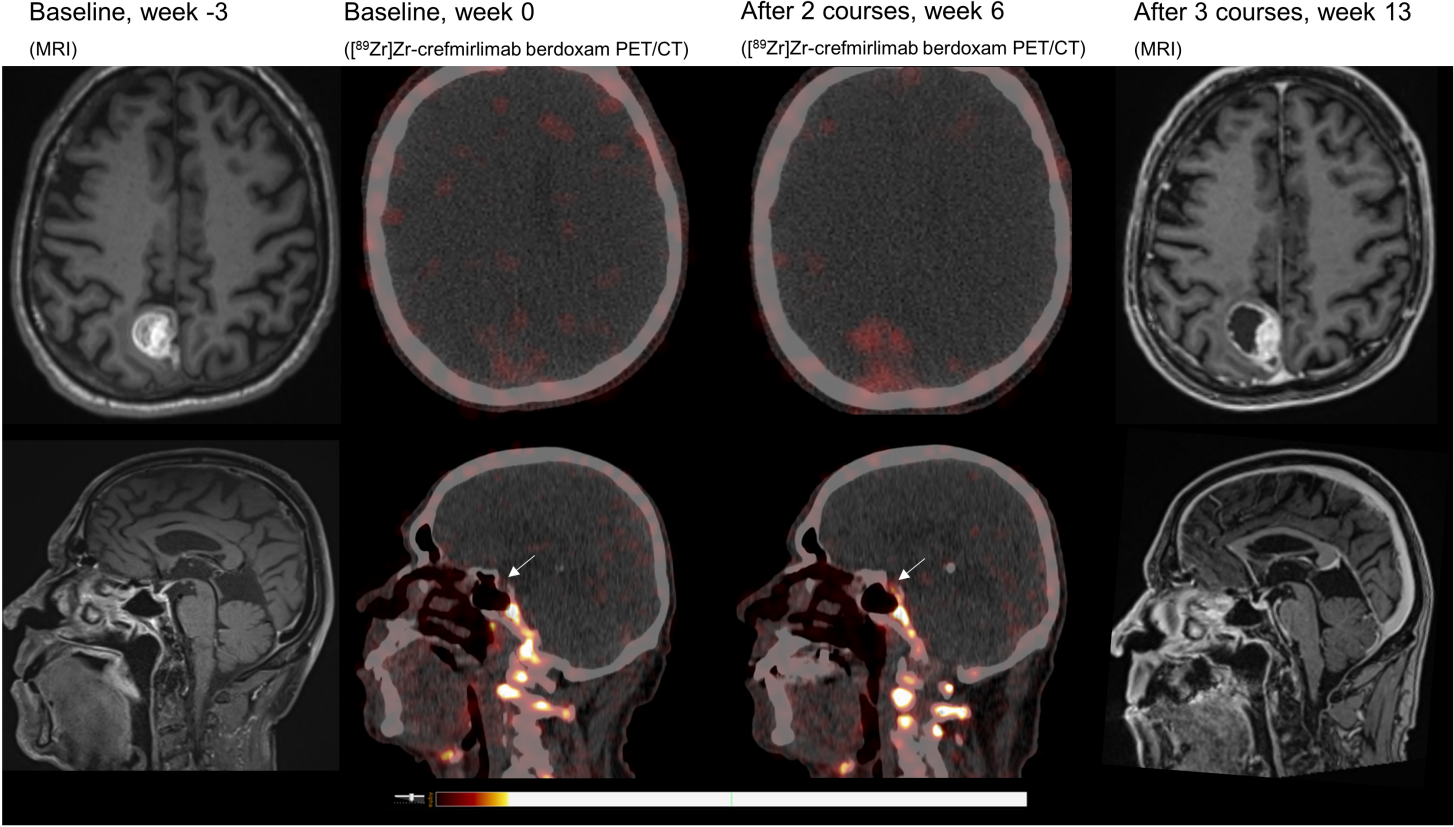
MRI and [^89^Zr]Zr-crefmirlimab berdoxam PET/CT scans of cerebral lesion and pituitary gland.

Retrospective analyses of the [^89^Zr]Zr-crefmirlimab berdoxam PET scan showed an increase in tracer uptake in the pituitary gland after two cycles of combination treatment, from maximum standardized uptake value (SUV_max_) 1.3 to SUV_max_ 3.7, suggesting treatment-induced increased infiltration of CD8+ T-cells (**Figure 1**). In parallel, the tracer uptake in the cerebral metastasis increased 2.8-fold, from (SUV_max_) 1.6 at baseline to SUV_max_ 4.5, suggesting increased CD8+ T-cell tumor infiltration. Imaging preceded the clinical presentation of ICI-induced hypophysitis by 8 days. Moreover, one day prior to the scan the patient was seen at our out-patient clinic and showed no symptoms of hypophysitis. On the day of the scan the patient had a normal potassium value and only mildly lowered thyroid hormone values (TSH 0.50 mE/l; FT4 8.7 pmol/l). No increase in tracer uptake was observed in downstream endocrine organs, ie. thyroid gland or adrenal glands (**Table 1**; **Supplementary Table S1**).

**Table 1:** Lesion size and tracer uptake measurements on baseline and follow up imaging.

	Baseline	Baseline	After 2 courses of treatment	After 3 courses of treatment
	Largest diameter, CT/MRI (mm)	Tracer uptake, PET (SUV_max_)	Tracer uptake, PET (SUV_max_)	Largest diameter, CT/MRI (mm)
Tumor lesion				
Cerebral	18.9	1.6	4.5	18.5
Gallbladder	43	2.9	3.6	39.8
Left maxillary sinus	17.1	2.2	1.5	15.1
Ampulla of Vater	non-measurable			
Organ system				
Pituitary gland		1.3	3.7	
Thyroid gland		3.1	1.5	
Adrenal gland		4.0	2.9	
Liver		13.6	15.5	
Bloodpool		1.2	1.6	
Spleen		61.1	60.1	
Bone marrow		14.6	17.1	

## Discussion

ICI-related hypophysitis is an infrequent but potentially serious adverse event that occurs more often in patients treated with combination immunotherapy compared to monotherapy (6, 7). Like other endocrinopathies, pituitary dysfunction is irreversible and patients require lifelong hormone replacement therapy. High-dose corticosteroids are rarely indicated and ICI treatment can be continued in most patients (8).

[^89^Zr]Zr-crefmirlimab berdoxam is a humanized minibody that targets the α-subunit of human CD8 (5) for the purpose of *in vivo* tracking of CD8+ T-cells, as main effector cells of anti-cancer immunity. Its capacity to predict response to checkpoint inhibition and detect toxicity is currently being investigated in a multi-center phase 2b study (NCT05013099) in which this patient participates. Similarly, CD8+ cytotoxic T-cells are suggested as mediators of ICI-related toxicities, however, reports on direct correlation are limited (9) as non-invasive tools to track CD8+ T-cells in patients were lacking. Conventional medical imaging as magnetic resonance imaging may reveal tissue changes commonly related to inflammation, e.g. swelling, edema and/or fibrosis (10) at clinical presentation, however, the role of anatomical imaging modalities in early detection is not investigated. As ICI-related toxicity is likely accompanied by other features of inflammation such as increased perfusion, this may also contribute to increased signal on PET. Although the increasing ratio of tracer uptake in the pituitary gland over bloodpool activity suggests accumulation of the tracer in the interstitial space, further research should confirm that the PET signal is due to CD8+ T-cell infiltration. In addition, the method of quantification of PET signal and definition of organ-specific threshold values associated with toxicity is currently under investigation.

This case demonstrates that CD8+ PET imaging has the potential to evaluate CD8+ trafficking to tissues at risk for ICI-related toxicity at early timepoints during treatment, and warrants further exploration of this modality in relation to management of severe ICI-related toxicity.

## Conclusion

Non-invasive CD8+ T-cell tracking in patients treated with immune checkpoint inhibitors revealed not only increased tracer accumulation in the brain metastasis, but also in ICI-related hypophysitis on a PET/CT scan made 8 days prior to onset of symptoms. These observations suggest that PET-based CD8+ T-cell trafficking in patients is a potential tool for early monitoring of ICI-related response and toxicity.

## Data availability statement

The original contributions presented in the study are included in the article/**Supplementary Material**, further inquiries can be directed to the corresponding author.

## Ethics statement

The studies involving human participants were reviewed and approved by METC Oost-Nederland. The patients/participants provided their written informed consent to participate in this study. Written informed consent was obtained from the individual(s) for the publication of any potentially identifiable images or data included in this article.

## Author contributions

All authors listed have made a substantial, direct, and intellectual contribution to the work and approved it for publication.

## Supplementary material

Supplementary Table 1 Tracer uptake in reference tissues on baseline and follow up imaging.
