# Supplementary material for: Case Report: Imaging immune checkpoint inhibitor-induced yin-yang effects in the brain
Source: Front Immunol. 2023 Jun 2;14:1199282. doi: 10.3389/fimmu.2023.1199282 (PMC10272360; doi:10.3389/fimmu.2023.1199282)
Supplement: Supplementary Table 1 — Tracer uptake in reference tissues on baseline and follow up imaging. [file Table_1.docx]

# Supplemental material to “Case report: Imaging immune checkpoint inhibitor-induced yin-yang effects in the brain”

Table S1.

Tracer uptake in reference tissues on baseline and follow up imaging

|  | baseline | after 2 courses of treatment |
| --- | --- | --- |
|  | tracer uptake, PET (SUVmax) | tracer uptake, PET (SUVmax) |
| Organ system |  |  |
| Lung | 1.8 | 1.1 |
| Colon | 20 | 11.2 |
| Stomach | 1.2 | 2.3 |
| Myocardium | 1.1 | 1.4 |
| Gluteus muscle | 0.4 | 0.7 |
